# After the Storm: Inter-disciplinary Dialogic Discourses With a Post-Fishing Community

**DOI:** 10.3389/fsoc.2020.00042

**Published:** 2020-06-30

**Authors:** Carol Stephenson, Fiona MacPherson

**Affiliations:** ^1^Department of Social Sciences, University of Northumbria, Newcastle, United Kingdom; ^2^Department of Art, University of Northumbria, Newcastle, United Kingdom

**Keywords:** public sociology, applied theater, dialogic discourses, post-industrial, inter-disciplinary

## Abstract

This paper is a critical evaluation of a unique approach to working with disadvantaged communities, which involves inter-disciplinary collaboration between an Applied Theater (AT) director and a sociologist. The application of the approach, in a community disadvantaged by the loss of industry, provides the case study basis for the evaluation. Between 2014 and 2017 community participants from Eyemouth, southeast Scotland, worked with an artistic team led by director Fiona MacPherson^1^, and a sociologist, Carol Stephenson, to develop a creative performance of the town's fishing disaster of 1881. This inter-disciplinary project was facilitated through dialogic discourses between community participants, AT director and sociologist in which the equalization of relationships, meaning-making and active listening were established as shared values and processes. The paper makes four claims. Firstly, sociological observation of the negotiation of the creative process revealed previously hidden and nuanced social interactions, which could later be examined in greater detail with the AT director and in focus group discussions with community participants. Second, the use of dialogic discourses in the critical appraisal of AT practice by the sociologist ultimately enabled the inter-disciplinary sharing of practice, ideas and theories that were mutually beneficial. Third, the creative process revealed insights into the lived experience of post-industrial communities and enabled public sociology discourse, which ultimately prompted social activism within the case study community. Last, while the inter-disciplinary approach is labor intensive and demands high levels of commitment to the shared values associated with dialogic discourses, it provides a new and innovative way of working with, and for, disadvantaged communities.

## Introduction

On October 14th 1881 the fishing fleet of Eyemouth, South East Scotland, driven by fear of starvation, set out as one. Unbeknown to the crews they were in the eye of a storm that would claim the lives of 189 men and boys. Women and children watched from the shore as their kin drowned in sight of land. The disaster left 93 widows and 267 children without fathers. The authorities sought to support the impoverished town by taking into care the children of those who had died. The collective endeavor of the people of Eyemouth ensured that this harsh charity was successfully rejected: not one child was removed (Aitchison, 2006).

Today Eyemouth is a post-fishing community that experiences many of the problems common to such communities.

In 2014, the Eyemouth Church Council made a complaint to the producers of a national tour, Follow The Herring (FTH), that incorporated the play Get Up and Tie Your Fingers (written by Ann Coburn): while FTH retold the story of the Eyemouth disaster, the tour had not visited Eyemouth itself. The decision not to perform in the town had been driven by a number of logistical considerations^2^. Following that complaint, AT director of the national FTH tour, Fiona MacPherson, worked with residents of Eyemouth to support them in a contemporary re-retelling of their story, using narration and song.

This further iteration of the project, Get Up and Tie Your Fingers Eyemouth (GUTYFE) provided a performative context for the inter-disciplinary collaboration of sociologist Carol Stephenson and MacPherson in the application of a unique approach to working with a post-industrial (PI) community, the critical appraisal of which forms this basis of the paper. The project took place over 20-month period and involved over 40 rehearsals and culminated in three iterations of GUTYFE^3^.

Stephenson had a long history of research in PI communities in relation to the conditions of their decline (Wray and Stephenson, 2012); female activism during and after the 1984/5 miners' strike (with Spence and Stephenson, 2006, 2007, 2009, 2012; Spence, 2019); and the value and importance of the performance of shared cultural and industrial heritage (Dodds et al., 2006; Mellor and Stephenson, 2005; Stephenson and Wray, 2005, 2017).

MacPherson is a theater director and academic whose work is informed by the principles of AT. She consequently values collaborative relationships between theater makers and community participants that are dialogic and democratic. She also recognizes these relationships and processes must be informed by a clear understanding of the socio/political context in which the creative work takes place: this prompted her collaboration with Stephenson.

The pair first collaborated when MacPherson invited Stephenson to support the development of The Awkward Squad (2012), a critically acclaimed piece of popular theater (Williams, 2012). The piece centered on the lives of three generations of women in a post-mining community. Stephenson's early involvement in the development of the storyline and engagement with actors in rehearsals provided sociological insight into female activism. To this point, the collaboration was a conventional arrangement as academic experts are often drawn into creative processes to provide background knowledge based on their own research. However, it quickly became apparent that a new type of opportunity was emerging. Following the knowledge exchange, Stephenson prompted MacPherson to justify creative decisions and interventions. MacPherson recognized the value of Stephenson's presence in the rehearsal, not simply because of her research-based knowledge but for the sociological skills of observation, criticality and the problematization and theorization of power. Stephenson's role had shifted from expert/information provider to curious critical friend and evaluator of creative process.

That early work, and the collaboration explored in this paper, was founded upon a shared commitment to liberation pedagogies that facilitate collective learning and creativity (Stephenson et al., 2014, 2016). The work in Eyemouth explored here is based on an examination of a series of integrated dialogic discourses between theater practitioners, academics and community participants. The theoretical foundations, explored in detail later, rest on dialogic discourse and pedagogic practices which forefront active listening and ethnographic approaches to the exploration of social meaning, culture and identity. Dialogic discourse refers to exploratory conversations between people of equal status that are characterized by mutual respect, acknowledgment of the validity of the knowledge base of others, intellectual openness and the possibility of critique and creative thought (Vygotsky, 1986; Freire, 2006; Barnes, 2008; Alexander, 2019).

The collaboration between MacPherson and Stephenson examined here is not the first instance of artists and academics working alongside communities in order to facilitate informal education. The Settlement House Movement began in the nineteenth century with similar ambitions and persists to this day. The Movement was founded on Christian principals and saw academics, artists and writers, establish houses where educators sought to “meet on equal terms” with the poor (Gilchrist and Jeffs, 2001, p. 10). While it is undoubtedly the case that working class people excluded from education were given meaningful opportunities and subsequently produced some remarkable work (McManners and Wales, 2008) the Settlement Movement is dogged by charges of paternalism (Popple, 2006, p. 107–121). Many other projects have been undertaken by academics, practitioners and activists motivated by participation in mutually beneficial and facilitated learning—indeed this is at the heart of the applied AT approach. However, the approach explored here is unique in that it *seeks to establish a triumvirate dialogue* between practitioners of two distinct disciplines and a group of community participants *throughout* the creative process in order to facilitate mutual learning, critical reflection and research.

The aim of this paper is to examine the degree to which this unique inter-disciplinary approach has the potential:

- To enable the critical evaluation of the practices and facilitation of the AT director—an aspect of the discipline which is underdeveloped (Mackey, 2016; Readman, 2018).
- To enable sociological research with disadvantaged communities which is beneficial to that community through the interrogation of, and engagement with, the creative performance of shared industrial heritage.
- To facilitate public sociology for social activism in a disadvantaged community.
- To examine the degree to which the approach might be transferable to other settings.

The paper begins with an examination of the ambitions and challenges faced by each discipline. Following this, the problems facing PI communities and specifically fishing communities, is explored along with an examination of the complex potential of the exploration of industrial heritage for such localities. Following that, the application of the collaboration between sociologist and AT practitioner in the development of GUTYFE provides the basis for the critical evaluation of the approach.

## Applied Theater and Sociology: Common Concerns and New Alliances

From its inception, sociology has been concerned with understanding and challenging social inequalities. Wright Mills (1959) conceptualized the sociological imagination as a quality of mind capable of empowering people to make sense of the interconnected nature of agency and wider structural forces: sociological thinking had the potential to be a catalyst for social change. Relatedly, the call for a public sociology (Burawoy, 2005) which connects the discipline and the sociologist to wider public audiences sees value in the expansion of the discipline beyond the narrow parameters of academia. However, public sociology is stymied in the age of neoliberalism where the role of structurally created inequalities are denied and replaced by explanations which forefront individual failings (Lawler and Payne, 2018). The consequence is the stigmatization of the dispossessed “left behind” by neoliberalism (Tyler, 2014; McKenzie, 2015; Thomas, 2015).

A range of sociological research methodologies have been applied to understanding PI communities (Waddington et al., 1991, 2001; MacDonald et al., 2013; Tyler, 2014; McKenzie, 2015). While the insights this research reveal are valuable, the views of those affected by the end of industry are, even with the best and most meticulous of research intentions, prompted, mediated and analyzed by researcher intermediaries. Community interaction and negotiation are rarely directly observed in the moment within which they occur. Activist sociologist (McKenzie, 2015) ethnographic work in a Nottingham housing estate is valuable, but rare given that it was possible only because of her long personal association with the area.

Sociologists face challenges in accessing and gaining the trust of disadvantaged social groups, and concerns about power imbalances and the problem of academic appropriation remain. Accordingly, while sociological research produces insights about the lived experiences of disadvantaged people, even when done with political and ethical sensitivity, the work does not necessarily result in those affected rethinking their social and political situation in relation to the structured nature of that injustice. To that end, sociological research can be politically and socially impotent.

By contrast, AT, which shares with sociology a commitment to social equality and cultural democracy, offers a dynamic and creative opportunity to engage with disadvantaged communities.

For the purposes of this paper, a brief introduction to the wider discipline of Applied Theater is appropriate. Applied theater, although not a fixed term, is broadly understood as an umbrella for a range of performance techniques and dramatic activity (Prentki and Preston, 2009, p. 2) which situates itself outside mainstream theater and performance contexts, located in a variety of “non-traditional settings and/or with marginalized communities” (Thompson and Jackson, 2006, p. 92). It is a field in which the discursive processes of theater making are as important as the product itself: AT “is a way of conceptualizing and interpreting theatrical and cultural practices that are motivated by the desire to make a difference in the world.” (Nicholson, 2014, p. 20). Its forms are many, amongst others, theater in education, prison theater, heritage theater, theater for development, theater for health education and, pertinent to this investigation, community based participatory theater.

The directorial practices and priorities of AT practitioners are as varied as the field itself. Each practitioner will adopt numerous “identities” (Readman, 2018). What they share is a commitment to be responsiveness and collaboration in order to enable participants to engage safely in the co-creation of community based participatory theater making. Each community of participants require specific rehearsal strategies and techniques appropriate to them.

Applied theater research (in relation to content, context and inspiration) and analysis of practice draws on a number of disciplines—notably in the social sciences, cultural studies, and education: AT is intrinsically an inter-disciplinary and hybrid practice (Nicholson, 2005, p. 2).

As AT seeks to “address something beyond the form itself in order to promote positive social processes within a particular community” (Ackroyd, 2000, p. 2), concern for community based learning is important. It is little wonder that AT frequently draws upon liberation pedagogies (Freire, 2006), which reject traditional teaching methods that rely upon the hierarchical transmission of knowledge in preference for a learner-centered pedagogy. AT practice embraces co-production strategies in community based participatory theater where community knowledge drives creative content and development. The utilization of liberation pedagogies, ensures that the ongoing negotiation of power, learning and knowledge (between practitioner and participant/performer) remains open to critical scrutiny. Consequently, AT offers participants the opportunity to increase and expand skills and confidence, connect and reconnect with their community, explore the past and/or hitherto neglected debates and to present, though performance, a public expression of place and community. It can shed light on the creative uniqueness of that community, challenging the standardization of cultural products and lastly, agitate for change through collective voice. Applied theater is, therefore, a cultural construct as well as a cultural product (Kershaw, 1992).

Within the practices and performance of AT, performance as research (PaR), offers opportunities for research for the AT practitioner and potentially for others (Kershaw and Nicholson, 2011, p. 63–64). However, PaR presents the practitioner with a number of significant challenges. Applied theater depends upon the practitioner's continual responsive engagement with the performer and consequently their perspective is reframed because of that interaction, challenge and recalibration. Applied theater often takes place over the course of long periods during which the practitioner, immersed in their work, is not easily able to reflect on or record practices and outcomes. Viewing a practical community theater project from a researcher perspective, the AT director is both the subject of the research, the analyst and the knowledge creator. Relatedly, the process of theater making and facilitation of community performance by experienced practitioners may come to be seen as “second nature.” Consequently, directorial processes in AT practice have rarely been the focus of systematic research (Mackey, 2016; Readman, 2018).

As the AT practitioner faces the challenge of how to evaluate critically his or her own practice, a research collaboration with a sociologist is a useful/logical step. After all, the work of the AT practitioner involves the engagement of social groups willing to discuss, explore, and evaluate social and political issues in a dynamic and creative manner. This offers opportunities to sociologists who face issues in engaging with disadvantaged social groups and non-academic audiences. There have been collaborations between the two disciplines in the past and these have tended to involve analysis of the emergence and nature of the theater form (Kim, 2019), the establishment of ideas or knowledge exchange (as in the Case with the Awkward Squad) or the evaluation of AT. Consequently, the role of the social scientist has been frequently confined to the evaluation of the impact or meaning of performance on the participants/audience, *outside of, prior to or after the event* rather than as part of an investigation into what *the creative process itself* might reveal about the lived experiences of social groups. A case in point is Walkerdine and Mackey's collaboration, Performing Abergavenny (Mackey, 2016, p. 6). Here, the social science contribution usefully validates a project in terms of the value of Arts funding, however the social science research analysis gives the AT director few, if any, insights into their practice and any wider sociological insights, which emerge in the creative process, go unrecorded.

A recognition of both the limitations and opportunities inherent within the two disciplines prompted MacPherson and Stephenson to investigate the potential of inter-disciplinary collaboration based on a common commitment to dialogic discourse through collaboration on GUTYFE.

## Post-Industrial Fishing Communities and the Problem and Potential of Shared Heritage

Post-industrial coastal communities in the UK have seen the loss of key industries of fishing, shipbuilding and mining, which has resulted in growing social breakdown. Consequently, they face some of the worst levels of deprivation in the UK, with high unemployment rates, particularly in the North East of England and Scotland (Centre for Social Justice, 2013; Corfe, 2017).

Eyemouth, the focus of this research, has seen a dramatic decline in employment associated with fishing during the past century. In the 1970s, over 50 large-scale boats anchored off Eyemouth harbor. After 2000, trawling for white fish declined dramatically. By 2020 only 20 local boats fishing for prawn, lobster and brown crab remained. (Richard Lawton, Eyemouth Harbor Master, February 2020)^4^.

This pattern is repeated across Scotland and its impact is more than economic. Williams (2008) argues that fisher folk conflate the declining health of the fishing industry with the “death” of community: loss of boat- building, empty harbors and the decline of fishermen has led to the subsequent decline in local spending power, an increase in substance abuse, and social isolation. In addition, the pressure to remain solvent and land acceptable legal quotas lessens the pleasure of fishing. That, and the disruption and/or eradication of intergenerational and extended family relationships founded on co-operative fishing practices, have had a negative impact on mental health and sense of identity as small scale fishing gives way to major commercial fishing enterprises.

Despite the stigmatization of PI fishing communities, respect for the “fishing way of life” continues to have resonance (Nadel-Klein, 2003), as mining life does for those living in post-mining communities (Mellor and Stephenson, 2005; Stephenson and Wray, 2005; Dodds et al., 2006; Dicks, 2008). However, a dependence on heritage as a route to community and economic regeneration is fraught with challenges.

Representations of fishing heritage has stimulated regeneration in post-fishing communities (Cornwall Gov Uk, 2018) through the establishment of tourist-based economies. This retelling of the past to external audiences is fraught with potential hazards. A significant issue is whose heritage is to be recognized. Versions of the past “from above” may deny diversity, complexity, and ambivalence (Rowbotham, 1992). Relatedly, if tourists seek to consume “authenticity,” newcomers to the community may be excluded from the collective celebration of their home. Far from building cohesion within traumatized communities, heritage may further divide communities. While the notion of hard-work and resilience, which typically defines the heritage industry's version of “fisher folk,” is not inaccurate, it depends on the maintenance of a shared identity which may be neither inclusive nor wholly authentic and which Dicks describes as the “view from the hill” (Dicks, 1999, p. 352). Nadel-Klein points to the irony of the survival of former fishing communities depending on their transformation into cultural show-cases of a particular version of heritage which, “selectively appropriates the past for the present providing a legacy of tradition, invented or otherwise” (Nadel-Klein, 2003, p. 174). While such a representation may, for some, reap economic rewards it is unlikely to offer a secure foundation for an inclusive sense of identity upon which a community can build a coherent vision for the future.

Despite these complexities, Nadel-Klein believes heritage is capable of capturing “multiple, contested, and mutually constituted meanings” and therefore heritage-led regeneration “may not exclude the locals after all” (Nadel-Klein, 2003, p. 211). While she is not specific about what this might look like, she agrees with Stephenson and Wray (2005, 2017) that celebration of industrial heritage can enable PI communities to hold on to a sense of who they are.

## Inter-Disciplinary Collaboration and Dialogic Discourses

In 2014, MacPherson directed Follow The Herring (FTH) in 12 coastal communities down the East Coast of Britain and, in doing so, sought to enable communities to engage with the collective and creative performance of shared fishing and heritage in a way in which Nadel-Klein and Stephenson and Wray believed possible^5^.

The University of Northumbria was commissioned to evaluate FTH for funder Arts Council England. This drew on wide range of methodologies but focused on the impact on audiences and participants after the event, as was the case with Performing Abergavenny (2018)^6^. Follow the Herring was hugely successful, in both the number of people it attracted and in terms of the overwhelmingly positive evaluation that validated the potential for the creative economy (Wilson, 2010). However, the evaluation's focus was on how participants felt about their involvement *separate to or after* the rehearsal or performance event. MacPherson believed opportunities to learn about PI communities, and about how AT worked within them, had been lost. Her close creative involvement with participants from post-fishing communities had revealed a series of social and political insights, interactions, negotiated meanings, and relationships, which she had not been able to capture and/or analyze given the demands of the tour and her role within it. Recognition of this missed opportunity prompted MacPherson to invite Stephenson to collaborate with her in GUTYFE and work with the people of Eyemouth.

GUTYF Eyemouth involved 60 community participants. The oldest performer was in their mid-80s, the youngest was 11 years old. They were residents of Eyemouth, other nearby coastal towns and outlying areas. Participants in the performance met regularly over a 20-month period: their commitment to the project was considerable. A majority had family connections to the fishing industry; some had worked directly in it. A majority of participants were lifelong residents of Eyemouth and some could trace their families back to the time of the disaster; others were relatively new to the area^7^.

From the outset, participants were informed that the project provided a research opportunity for MacPherson, as she would use the views and actions of participants and the observations and insight of the sociologist Stephenson to reflect on her practice. The role of Stephenson in rehearsal and performance was made clear—to act as research collaborator with MacPherson—she would not directly intervene during rehearsal or performance events. However, in the focus groups that would follow the June 2016 performance, there would be an opportunity to talk directly with Stephenson about her observations of their work, the discipline of sociology and her research in PI communities similar to their own.

The principles of dialogic discourse underpinned all the conversations that took place during GUTYFE and in the reflections that followed. Dialogic discourse refers to a form of communication, which involves extended conversations founded on the equalization of relationships. Those involved in these conversations recognize the value of the ideas, knowledge and skills of others. It seeks the establishment of deep, evolving understandings through a process of respectful questioning and listening. Each participant in the discourse has the opportunity to challenge and learn from others in order to reveal new understanding and potentials. As Bakhtin claims, “it educates each side about itself and about the other, and it not only discovers but activates potentials. Indeed, the process of dialogue may itself create new potentials, realizable only through future activity and dialogue” (Morson and Emerson, 1990, p. 55).

Alexander (2008) identifies five characteristics of dialogic discourse: they must be collective, reciprocal, supportive, cumulative, and purposeful. Barnes notes the importance of listening for those who are part of the dialogic group and points to the importance of exploratory talk which is hesitant and incomplete and which enables ideas to be tested and re-formed through a respectful process of speaking, questioning and listening (Barnes, 2008, p. 5). Dialogic discourse is typically associated with radical pedagogies, however it has potential within social research: if, as Vygotsky (1986) suggests, conceptualized learning is a social process of change which occurs because of dialogic discourse, then the creative practices and principles of AT provide a context for the emergence of new meaning and knowledge.

The inter-disciplinary dialogic discourse approach used in GUTYFE involved a series of overlapping and interrelated conversations. At the outset of each discourse, the value of the knowledge and experiences of all parties was reiterated. Three dialogic discourses formed the basis for the approach:

(1) Inter-disciplinary (AT practitioner with sociologist).

(2) AT Practitioner with Community Participant: (observed by the sociologist).

(3) Sociologist with Community Participant Focus-Group (observed by the AT practitioner).

These were cumulative and dynamic in nature: issues and knowledge that emerged within them informed other discussions and were discussed, and clarified during the development, rehearsal, performance, and reflection of the project. Here we consider each dialogue in turn to reveal what they made possible.

Inter-disciplinary (AT practitioner with sociologist) these dialogic discourses were constant throughout the creative process. Stephenson and MacPherson met a minimum of twice a week over a twenty-month period, each time for a minimum of an hour. Both parties agreed notes from each discussion.

These discussions were dialogic in nature as they were exploratory and based on reciprocal respect of experience and knowledge. In addition, they focused on self-reflection and a mutual commitment to identify the intersections between the two disciplines.

To illustrate, one of the issues raised by Stephenson related to MacPherson's approach to active “listening.” Here MacPherson discusses how dialogic practices were embedded in her AT work with GUTYFE and how and why the act of listening became the primary consideration of the rehearsal process.

*CS : Explain to me how dialogic discourses are achieved and why you are so concerned with listening*.
*F.Mac: Ok, for many years, I felt….that my role was to encourage the* telling*[MacPherson's emphasis] of stories… creating a place in which group members felt empowered to speak. Making space for new voices, often traditionally silenced, to be heard. Recently I've realized the importance of listening…. listening necessities different qualities of engagement, social, political and artistic, and by prioritizing listening over speaking in the rehearsal room, I've found it not only challenges received notions of ‘quality’ and ‘authenticity’ and ‘talent’, it opens up the space to concentrate more closely on what is being said, rather than how the words are uttered……as a result a qualitative shift occurs. So we move away from the traditional skills of acting and ‘actorliness’ and concentrate more on the story they are telling. So, then it is my turn to listen to them…. about the town in which they live, their past and their perceptions of the impact this tragedy had on the community left behind*.
CS : So, how was that done?
*F.Mac: Ok, when I met the narrators [10] they were concerned about learning ‘all those lines!’…. I knew that learning the lines and pretending to be someone else whilst saying them was an unnecessary pressure…a distraction we didn't need. So I told them….they would read their story in performance, and they wouldn't need to pretend to be someone else, they would just ‘be’ themselves. The playwright had given permission for us to further adapt the text …it was therefore fluid and I encouraged them [performers] to cut and change where necessary. This editing was done through a process of active listening. During an early rehearsal, the narrators carefully read the piece aloud, taking turns to read different lines and speeches, those not reading were asked to listen closely to what was being said. It was an intimate exercise that took place over a couple of hours. Each line was interrogated by the group in relation to how they heard it….they highlighted words they enjoyed saying, they were asked to identify any words that felt not quite right, ‘not quite Eyemouth’*.
CS : What was the point of this? What was the outcome?
*F.Mac: The aim here is about sharing power, it is about attempting to make the rehearsal room discursive, it was about turning it into a respectful, purposeful conversation, a dialogic discourse with a clear focus – to curate an authentic story about the disaster for a contemporary audience from their town….it gave them the power to make qualitative decisions about the text and who felt comfortable saying what …they are not pretending to be actors, they are representing their community as they saw fit. Here we are creating an environment where they speak as themselves with a confidence because it is their story…I am not interested in them ‘learning acting skills’; but in helping them to stand there authentically with a confidence that is different from acting, that is being…. I am interested in Nancy's (**2002**) theories of listening*.
CS : Did you share Nancy's theories with the community participants?
*F. Mac: Err…I didn't talk to them about Nancy specifically but I did talk always about listening and listening techniques were embedded in what we did. But I didn't really think about how his theory could frame my practice until after the first performance. It wasn't until you made me start to theorise around my practice.…in the next stage of this project will be to explore the act of listening for information, for making meaning and for sharing with their audience… so it is a practical introduction but I would talk then directly about Nancy, his terms etc. I won't hide him*.

This interaction between sociologist and director involved the sharing of established knowledge and the emergence of new understandings: It enabled MacPherson to critically reflect on her initial suggestion that “instinct” shaped her approach to listening in the context of AT. Stephenson challenged the reference to “instinct”: MacPherson accepted that in reality years of theoretically, politically and socially informed experience and knowledge informed her practice. MacPherson's acceptance prompted a more detailed reflection on the theory underpinning her work. One important source was Jean-Luc Nancy's theorization of listening, which draws a distinction between *entendre* and *ecouter*—both French verbs, to listen. For Nancy while e*ntendre* refers to listening in order to identify meaning, the term *ecouter* refers to a more embodied listening, relating to emotional significance, a human listening. In addition, Nancy refers to *renvoi* (resonance) within listening—the return on the entendre and ecouter. Renvoi involves the sending and re-sending of communication between performers and between performers and audiences. GUTYFE participants engaged in a range of exercises, which sensitized them to different ways of listening, and reading returns on their communications (an example of the impact of this features in the next section). Sociologically, Nancy's work offers a reminder—if one is needed - that social exploitation and disadvantage has material and personal/social-emotional manifestations and legacies and both are legitimately the concern of the discipline. Theories of AT applied theater provide a foundation for a critical and complex understanding of how collective emotion is communicated and, intriguingly, the practice of AT provides a platform for that communication and one in which the sociologist can be engaged.

## At Practitioner With Community Participant Discourses (Observed by the Sociologist)

These dialogic discussions took place between the AT practitioner and community participants during the development and rehearsal of performances some of which were observed by Stephenson. Consequently, the sociologist was able to see the sharing of existing knowledge and the development of new understandings that emerged “in the moment” in which they occurred. These insights were discussed later in inter-disciplinary conversations and in sociologist-led focus groups with community participants.

It became apparent in observation of rehearsal discussions that, for those who had a long-standing association with fishing in Eyemouth, the disaster had ongoing social and personal importance. The events of the day were well known. This were a source of both pride *(“Eyemouth crews went out as one - that was the Eyemouth way”*), and sadness: the state had failed them and the struggle to keep their children had been immense.

At a personal level, some participants could trace their family back to the disaster and that was a source of pride and sadness. Participants commented that one family had a relation who had been at a wedding on the day of the disaster and so had not gone out. Some of those that had lost relatives had not been able to speak to him or his family; his survival meant that he had somehow “broken rank.” The sharing of grief and hardship made the community's survival possible. Observation of discussions that emerged in the development of the performance demonstrated how that loss and hardship had cast a shadow over the town that was both economic and emotional. One of the older female participant's spoke of childhood memories of the specific impact borne by fisher wives: “*they were always working, always in long black dresses bent over, baiting the lines”*.

It became evident that that the performance had provided a lens through which aspects of community life, which had not previously been directly expressed and explored, were raised. Specifically, this related to the importance of the sacrifices people had made because of association with the fishing industry, the respect for (and loss of) past collective ways of living. This came into sharp relief when participants empathized emotionally with the loss and sacrifice associated with the disaster and consequently chose to take control of the performance.

To illustrate, in one rehearsal narrators relayed verbatim the words of women who had lost their menfolk: “…. *and what do they write about us, the women left behind*?^8^” The script required the narrators to move from reporting in the third person to talking in the first: to move from storyteller into a character. However, one narrator chose spontaneously to put down her narration book and took center stage to speak these lines neither as a narrator nor as a character but as herself, as a mother speaking directly to an imagined audience about loss. This emotionally charged moment, that affected those that witnessed it, was incorporated into the final performance.

A further illustration came when GUTYFE were asked to perform 5 minutes of their performance at the unveiling ceremony of a commemorative sculpture, which named those lost in the disaster, to mark the 135th anniversary of the disaster. In the original GUTYF script, only the names of the boats were mentioned but the GUTYFE participants insisted that the names and ages of those lost were included in their version. The request for 5 min of the performance gave the participants new authorship responsibilities, which they took very seriously. In an inter-disciplinary discussion, MacPherson explained:

*So which 5 minutes? I said Ok try this: we start a name but when the surname is said, it is overlaid by the Christian name of the next person - so it is like waves crashing on the shore one on top of the next*.

When this was attempted in the rehearsal, participants rejected the plan as the full names of these who had died were obscured. Performers knew that the descendants of those who had died would be waiting to hear family names loud and clear in and their entirety:

Jimmy: *This is not right! This is not working. We've got to hear the names!*
F Mac: *If we say all the names, we will be in to 9 minutes…*.
Jimmy: *Let them bloody wait*!

Here we see the point at which the group are unprepared to compromise, defending what is most important to them. MacPherson later commented, in relation to the theories of inter-active listening explored above, on the profound emotional impact of that decision on the performers and audience when the names were read in full:

*It was arguably the most powerful moment I had witnessed….the performers knew this because they felt it, they were louder and bigger and more authoritative than they had been before, they embraced it….as those names were hitting that audience and the recognition was setting in, you could have heard* a *pin drop…it was a moment in which the group were unified*.

Here we see the importance of Nancy's distinction between entendre and ecouter and renvoi: the names are communicated, emotionally felt, shared and returned. This is a shared, dynamic experience that is both cognitive and emotional. This theory and practice has potential in relation to the exploration of the sociological concept of social haunting which Stephenson examined with MacPherson in inter-disciplinary discussions. Social haunting, is the view that communities are affected by structures of feeling, collective memories and meanings, typically associated with collectively experienced trauma. These are ghostly as they are not necessarily directly acknowledged but persist in the culture and collective consciousness of traumatized communities (Gordon, 2008). Such ghosts can be realized through creative lenses—GUTYFE had become such a lens—and realization can enable a community to move on (Bright, 2018; Spence, 2019). Stephenson argued that from a sociological standpoint the disaster, and the hardship that followed, and indeed more recent processes of de-industrialization, cannot be understood as merely distant historical events. The nature of historical trauma and the degree to which that has been collectively realized has a bearing on how well social groups deal with contemporary challenges and injustices. In the focus groups that followed concepts of loss and abandonment became an overarching leitmotif when community performers explored both the disaster and the challenges facing Eyemouth in the contemporary era.

## Sociologist With Community Participant Focus-Group (Observed by the at Practitioner)

Two focus groups occurred following the June 2017 performance of GUTYFE, one with narrators, the other with the choir. In total 30 of the 70 participants attended.

The focus groups allowed for a direct discussion about events and issues observed by the Stephenson in rehearsals and performance and provided an opportunity to discuss sociological concepts, research and practice. At the same time, they gave MacPherson an opportunity to listen to the discussion and reflect on her practice. They also provided an opportunity to talk directly about sociology, sociological skills and research, and existent sociological knowledge and concepts associated with PI communities. Questions typically began with ‘*sociologists are interested in*.’.

Focus group dialogic discussions were affected by the shared experience of engagement with GUTYFE and by the AT application of an approach to listening which had embedded the principles of dialogic discourse. Consequently, the discussions differed from usual focus groups in three key ways. Firstly, the participants had shared experience of the project, which provided a frame of reference through which to consider their community: participants drew attention to rehearsals/performance, decisions and disagreement, things they had tried and which had worked or had not to illustrate their points. Consequently, they were not simply responding as individuals to the questions set by Stephenson, they were involved in a reflective and well-developed dialogue with each other, which was established over time in their work with MacPherson. Secondly, Stephenson had witnessed group interactions and had talked to MacPherson, so had knowledge of how issues were raised and addressed and was able to raise some of these directly with participants. Thirdly, following MacPherson's use of a dialogic approach within the creative process which fore-fronted the exploration of ideas, the participants were noticeably engaged in active listening, prepared to take risks and being open to ideas.

Two inter-related themes emerged in the focus groups. One was in relation to the importance of the message in their shared industrial heritage, specifically in relation to the resilience of past generations. Attached to that was the value of collective focus and communal conversations. The second theme was of a sense of loss and abandonment, which was a leitmotif within the discussion: the loss of easy familiarity with others; of common purpose (fishing); of the absence of state support for fishing and frustration with Brexit progress; the loss of communal spaces; of grown up children; of pubs, shops and collective celebrations.

In focus groups discussions participants claimed that GUTYFE, because of its theme of the Eyemouth fishing disaster and resilience and its form (by the townsfolk, for the townsfolk) had drawn in people, both as participants and audience, who would normally eschew such events. Consequently, the project had widened debate and begun new and unexpected conversations. To illustrate, one female participant commented that after the performance an elderly local fisherman—“*A man's man, sometimes he will speak sometimes he will no*” - had crossed the street to speak to her:

“*It was the Monday after the shows, I was walking along the harbour after being out in my boat when I saw an old Fisher I know – a bit of a bluff gruff chap – a lovely singer though. He didn't want to be part of the Get Up choir though. He crossed the road to talk to me – which is a bit of a surprise as he's more likely to pass on by, but he stopped and said, ‘I was there, Saturday night I was there! And I was in tears more than one once!’. very emotional*”.

It was felt that the project had provided a common purpose through which new understandings and relationships could emerge. To illustrate, as GUTYFE is delivered in a Scottish Dialect the story could potentially have been exclusive to those who were “long-term” Eyemouthians. However, this proved not to be the case, largely because of MacPherson's careful approach to inclusion, which allowed participants opportunity to collectively reflect on and negotiate involvement. Participants outlined a number of positions in relation to their legitimacy to contribute:

Christine: *Err well …I wanted to be involved but I decided that as I didn't have the accent, I would be involved but not as a narrator, I would be in the choir*.
Amanda: *Well no, I think you should. When I heard about this and I wanted to do it so that I could be more involved, more included ….but I was like ‘Is it right and proper as an outsider to be doing that?’ and in the end I was just like, ‘Well actually I don’t care, I want to do it so I'm doing it'!.Yay', I've come in here and I feel a bit more, yeah, good this is a community that I'm getting to know already, so….and yeah, it sounds a bit sort of pious, but, I like to, if I'm living in a place, I like to give something to the place. I'm not interested in sort of being an incomer who then goes ‘Eyemouth, I don’t care about your history!'. No! I do, I do, to me that's part of living in a place!”* (Woman in her thirties with young family, new to the locality).

Despite differences in approach, each respondent was self-reflective, each had wished to give something to the community and each listened to the position of the other with patience: one had been self-censoring; the other has seen the GUTYFE as “a way in.” In the same focus group, Diana, a long-standing female resident spoke of their frustration that: “*Incomers don't want to know anything about the community; they don't want to join in'*. After listening thoughtfully to the reflections of those new to the community, Diana said she had not thought of her new friends as “outsiders.” The project had brought her face to face with some who had felt they might be seen in that way but had chosen to find a way to participate that was acceptable to them. Subsequently new relationships had been forged, prejudices overcome. MacPherson later acknowledged that but for GUTYFE these discussions might never have been aired directly. Her view was that this collective willingness to engage with and explore the standpoints of others stemmed directly from the relationships which had emerged as a result of the practices associated with dialogic discourse which formed the basis for the AT approach and the lens through which they explored the story.

A film was made of the final performance of GUTYFE^9^ in April 2017 and shown to participants in 2018. At that point, MacPherson asked community participants if there was an appetite for a new piece, which focussed on contemporary Eyemouth. The response was positive. MacPherson has since secured funding from Creative Scotland. A company (Berwickshire Coastal Arts—One Coast, Many Voices) has been formed to support this work. While the objective of this next stage is to improve access to, participation in and enjoyment of the arts, the impact of public sociology is evident: participants have undertaken training workshops and, since January 2020, have carried out qualitative research interviews within their own community. The participants have chosen to engage in discussion with social groups that had been previously under-represented in the GUTYFE: young mothers, teenagers, fishermen, the Lifeboat Crew, The Harbourmaster amongst others. The material the group gathers will form the basis for ongoing dialogic discourse between sociologist, AT director and the community in the next stage of the telling of the Eyemouth story. Community participants have determined that this stage will involve a creative focus on the sociological themes explored within the development and performance of GUTYFE. These relate to belonging and not belonging; immersion in a new community and making a new life; re-connecting, returning and re-immersion in the community; building, a business, a life, a family; change in the economy, the area, harbor, family; the pull of the sea/home; the impact of tourism, climate change, the weather.

## Conclusion

Before examining the achievements and potential of this inter-disciplinary approach, it is important to acknowledge the challenges associated with it and that some aspects of it were not fully realized.

One key challenge was that while it delivered rich data and reward, it was both time consuming and labor intensive. Stephenson and MacPherson met twice weekly to share discipline-based reflections on its development, but it was not practically possible for Stephenson to be present at all rehearsals over a 20-month period. Future applications of the approach would benefit from the full-time focus of a sociologist, or indeed, a team.

A second was that three-way dialogic discourse “in the moment” in which insights were revealed did not take place for a number of reasons. While observation of the rehearsal and performance enabled Stephenson to see interactions in the moment of their airing/negotiation/resolution, she chose not to intervene immediately to question those involved: it was reasoned that this would have been intrusive and disrespectful. Stephenson reflected on observations until an opportunity to talk directly to MacPherson arose (two hours after rehearsal; 1 day after the final performance) and with community participants in focus groups (10 days after rehearsals).

A further limitation, thus far, was that while Stephenson discussed sociological ideas and questions with the participants she was cautious in introducing theory into the conversations. There was concern that this had not been invited by the participants and might therefore interrupt, divert or inhibit dialogic discourses: it was felt that in keeping with a dialogic approach these should be introduced if and when they were valuable to the issues raised by the community participants. Similarly, when MacPherson discussed issues relating to theoretical aspects of AT practice with participants, she did so on a “need to know” basis. Many of the theories underpinning her practice were not widely discussed: she reasoned this might detract from the spontaneity, focus and enjoyment of the work (see above, her concern about the potentially negative impact of “actorliness”). While a relationship of respect and trust developed between community participants and MacPherson because of collaboration in the creative process, Stephenson's role was one of observer and focus group lead, and she remained something of an outsider in the creative processes. Nevertheless, a platform of trust was established with the participants upon which a more detailed examination of the sociological imagination, sociological and theory can take place. In the next phase, Stephenson will engage in discussion about the meaning of the material gathered by community participants and provide a more explicit social, political and theoretical context within and throughout the creative development of the work based on that material. The intention is to pursue a triumvirate conversation (three way and equal) in relation to theory and practice between community, AT practitioner and sociologist in all stages of the creative development, rehearsal and performance of the telling of the contemporary story of Eyemouth.

Nevertheless, the GUTYFE demonstrated potential in relation to four important areas. These relate to the sociological research potential of collaboration with creative practitioners and processes in work with disadvantaged communities; the mutual benefit associated with inter-disciplinary critical appraisal of AT practice; the actual and potential of a platform for public sociology; the transferability of the approach.

The approach used in Eyemouth is a way of working with, and for, communities, which delivers the combined benefits of the two disciplines, and a strategy for creative and critical reflection on those disciplines. The dialogic discourse and interrogation of AT practice and the infusion of sociological ideas, theories and debates into the creative process shaped and enhanced the creative practices of the AT director and enabled her to critically reflection on practice. In addition, and perhaps most important sociologically, the collaboration also offered a unique research method. Sociological observation of the negotiation of understanding and meaning in the creative process exposed previously hidden and nuanced social interactions, which would not have been revealed through qualitative research strategies previously applied within such communities. In GUTYFE, the moment of creative process became a conduit for shared emotion and previously hidden meaning-making. If, as Gordon (2008) and Bright (2018) suggest, past problems faced by communities are connected, even if unconsciously, to contemporary problems then this inter-disciplinary approach offers not only an opportunity to make visible and explore those traumas creatively but for them to be seen, understood and directly discussed sociologically.

One of the key aims of the work was to explore how AT practitioners might benefit from the critical appraisal of the observation and intervention of a sociologist. Ultimately, inter-disciplinary discussion, observation, and self-reflection of the process proved to be mutually beneficial for both practitioners. The observation and investigation of AT practice and theory proved valuable from a sociological point of view, particularly in relation to those associated with the theorization and application of AT methods associated with listening and the potential of such a strategy for the exploration of social haunting associated with disadvantaged communities.

The third issue arising from this work relates to the degree to which this collaboration provided a platform for public sociology. A range of sociological issues were discussed—demographic change, loss of public spaces and resources, the absence or silencing of some stories, the impact of economic vulnerability on community cohesion. The curiosity of sociologist who found the town of interest and raised sociological and question prompted the participants to conduct their own social research to understand better the concerns facing the town in the contemporary era. The ongoing commitment of MacPherson to provide support for future performances provides an outlet and audience for the communication of those concerns.

A crucial question for this critical appraisal of this inter-disciplinary approach is the extent to which it is repeatable: this paper highlighted a number of conditions for the success of this approach.

An important condition was the political sensibilities of MacPherson and Stephenson, which directed them toward radical pedagogies within their disciplines: this was fundamental to the success of the approach. The Eyemouth project was not simply a collaboration between a sociologist and a theater maker, but between two who shared a common interest in how their disciplines could support the development of new, improved relationships and conversations that might facilitate collective creative social awareness and potentially social change.

A second was that the community of Eyemouth *invited* the involvement of the AT practitioner to support them in the creative telling of their town's heritage. This enabled MacPherson to invite legitimately the participation of the sociologist. In addition, it provided the basis for the equalization of relationships as it gave the participants control: the work had not been forced on the community.

A third important condition was that the content of the project had meaning for the community. The social and emotional draw associated with the exploration of shared heritage should not be underestimated. In Eyemouth, the project's content drew in many who would not otherwise have sought to engage with others at a creative level. Yet at the same time, the universal story of suffering and redemption allowed so-called “outsiders” to find meaning in the performance. Interest in shared industrial heritage continues to hold power either as a source of communal pride and/or as a conduit for the exploration and exorcism of past and contemporary injustices. In the case of Eyemouth, the trauma is not simply that of the 1881 disaster, but the loss of industry, way of life and identity associated with the decline of fishing and the discussion of the historical disaster became a way of talking about the present.

In the case examined here the motivating focus was shared industrial heritage; however, any shared collective experience or memory, which has meaning for a community, might usefully provide the basis for this approach. It would be wrong to suggest that communities are only defined by their past, particularly if that past is traumatic, but similarly wrong to suggest that past events have no meaning in the contemporary context. Gordon (2008) and Bright (2018) work on social hauntings suggest that only through the realization of trauma can communities move on: communities have a right and need to acknowledge the trauma associated with injustice. The inter-disciplinary approach used here, and the creative lenses it provided for the artistic and sociological exploration of shared trauma, provides a strategy for such an exploration elsewhere.

## Data Availability Statement

The datasets generated for this study will not be made publicly available to protect the identities of participants.

## Ethics Statement

The studies involving human participants were reviewed and approved by University of Northumbria, Newcastle. The patients/participants provided their written informed consent to participate in this study.

## Author Contributions

CS and FM contributed equally to the design of the research project, to the implementation of the research, to the analysis of data, and to the writing of their manuscript. All authors contributed to the article and approved the submitted version.

## Conflict of Interest

The authors declare that the research was conducted in the absence of any commercial or financial relationships that could be construed as a potential conflict of interest.

## Footnotes

^2^MacPherson was artistic director of FTH, a multi-arts touring production. The project was funded by the Arts Council England's (ACE) Strategic Touring Programme.

^3^Get Up and Tie Your Fingers Eyemouth: Eyemouth Parish Church April 2016. Get Up and Tie Your Fingers Eyemouth: Performance for the Dedication Service of the Widows and Bairn's sculpture, The Bantry, Eyemouth October 2016. Get Up and Tie Your Fingers Eyemouth: National Storytelling Festival—Tradfest Edinburgh April 2017.

^4^The town's fish processing industry (involving the export of live langoustine and shellfish) faces jeopardy as fear of friction in export trading following Brexit has prompted local employers to consider relocation to mainland Europe (Newsnet, 2019, Scotland, 2018).

^5^It involved a professional cast and 12 community choirs in a fictional drama, written by Coburn (2001). The community involvement was primarily as singers, performing a contemporary choral song cycle, composed by Karen Wimhurst. The play focused on the lives of fishing lasses and on the Eyemouth disaster. The appetite for community arts which reflect on fishing heritage proved considerable: 5,398 people attended performances of the play associated with the tour and 18,224 visited the exhibition of creative work. In the course of FTH almost six and a half thousand people directly participated in either collective knitting or the choral performances.

^6^The evaluation was comprised of site visits, audience and participant surveys, interviews and focus groups with those managing, delivering and participating in the project. It utilized participant diaries, audience comments books and exhibition feedback slips. Online participant and audience surveys were used to quickly capture experiences and views. The Participants Survey consisted of 37 questions covering, inter alia, demographic information; levels of participation in other cultural activities; health, wellbeing and individual circumstances; and experiences of participating in FTH was circulated to all participants and through Facebook and Twitter. In total, 86 participants and 146 audience members completed the surveys (Northumbria University, 2015).

^7^Informed consent was sought from community participants and comments were anonymized. The research was given ethical approval by the University of Northumbria.

^8^Reported The Berwickshire News Oct 1881.

^9^The National Storytelling Centre, Edinburgh (Netherbow Theater).

## References

[B1] Ackroyd J. (2000). Applied theatre: problems and possibilities. Appl. Theatre Res. 1, 1–13.

[B2] Aitchison P. (2006). Black Friday. Edinburgh: Birlinn Limited.

[B3] Alexander R. J. (2008). Culture, dialogue and learning: notes on an emerging pedagogy, in Exploring Talk in School, eds Mercer N. Hodgkinson S. (London: Sage, 93–114.

[B4] Alexander R. J. (2019). Dialogic pedagogy in a post-truth world, in The Routledge International Handbook of Research on Dialogic Education, eds Mercer N. Wegerif R. Major L. (London: Routledge, 672–686.

[B5] Barnes D. (2008). Exploratory talk for learning, in Exploring Talk in School, ed Mercer N. Hodgkinson S. (London: Sage, 1–17.

[B6] Bright G. (2018). A chance to talk like this: gender, education and social haunting in a UK coalfield, in Education and Working Class Youth: Reshaping the Politics of Inclusion, eds Simmons R. Smyth J. (Cham: Palgrave MacMillan, 105–129.

[B7] Burawoy M. (2005). For public sociology. Am. Sociol. Rev. 70, 4–28. 10.1177/000312240507000102 15926908

[B8] Centre for Social Justice (2013). Turning the Tide: Social Justice in Five Seaside Towns. The Centre for Social Justice (CSJ).

[B9] Coburn A. (2001). Get Up and Tie Your Fingers. London, UK: Oberon.

[B10] Corfe S. (2017). Living on the Edge: Britain's Coastal Communities. The Social Market Foundation (SMF).

[B11] Cornwall Gov Uk (2018). Available online at: www.cornwall.gov.uk/environment-and-planning/strategic-historic-environment-service/heritage-led-regeneration

[B12] Dicks B. (1999). The view of our town from the hill: communities on display as local heritage. Int. J. Cult. Stud. 2, 349–368. 10.1177/136787799900200305

[B13] Dicks B. (2008). Performing the hidden injuries of class in coal-mining heritage. Sociology 42, 436–452. 10.1177/0038038508088824

[B14] Dodds L. Mellor M. Stephenson C. (2006). Industrial identity in a post-industrial age: resilience in former mining communities. Northern Econ. Rev. 37, 77–91.

[B15] Freire P. (2006). Pedagogy of the Oppressed, 30th Anniversary Edition. New York, NY: Continuum.

[B16] Gilchrist R. Jeffs T. (2001). Settlements, Social Change and Community Action: Good Neighbours. London, UK; Philadelphia, PA: Jessica Kingsley Publishers.

[B17] Gordon A. (2008). Ghostly Matters: Hauntings and the Sociological Imagination, 2nd Edn. London: University of Minnesota Press.

[B18] Kershaw B. (1992). The Politics of Performance: Radical Theatre as Cultural Intervention. London: Routledge.

[B19] Kershaw B. Nicholson H. H. (eds). (2011). Research Methods and Performance. Edinburgh: Edinburgh University Press.

[B20] Kim K. -M. (2019). A trend in theatre programmes: the case of the Seoul Metropolitan Government. Am. J. Appl. Sociol. 1, 1–11. Available online at: http://acascipub.com.com/Journals.php

[B21] Lawler S. Payne S. (2018). Social Mobility for the 21st Century: Everyone a Winner? London: New York, NY: Routledge.

[B22] MacDonald R. Shildrick T. Furlong A. (2013). In search of 'intergenerational cultures of worklessness: hunting the yeti and shooting zombies. Crit. Soc. Policy 34, 199–220. 10.1177/0261018313501825

[B23] Mackey S. (2016). Applied theatre and practice as research: polyphonic conversations. Res. Drama Educ. 21, 478–491. 10.1080/13569783.2016.1220250

[B24] McKenzie L. (2015). Getting By: Estates, Class and Culture in Austerity Britain. Bristol: Policy Press.

[B25] McManners R. Wales G. (2008). Way to the Better: The Spennymoor Settlement. London: Gemini Productions.

[B26] Mellor M. Stephenson C. (2005). The durham miners' gala and the spirit of community. J. Commun. Dev. 40, 343–352. 10.1093/cdj/bsi020

[B27] Morson G. S. Emerson C. (1990). Mikhail Bakhtin: Creation of a Prosaic, 1st Edn. California, CA: Stanford University Press.

[B28] Nadel-Klein J. (2003). Fishing for Heritage: Modernity and Loss Along the Scottish Coast. London: Bloomsbury Publishing.

[B29] Nancy J.-L. (2002). Listening. Translated by Mandell C. 2007. New York, NY: Fordham University Press.

[B30] Newsnet. (2019). Available online at: https://newsnet.scot/news-analysis/is-eyemouth-facing-a-brexit-employment-tsumani (accessed December 8, 2018).

[B31] Nicholson H. (2005). Applied Drama: The Gift of Theatre. Basingstoke: Palgrave.

[B32] Nicholson H. (2014). Applied Drama: The Gift of Theater, 2nd Edn. New York, NY: Palgrave MacMillan.

[B33] Northumbria University (2015). Follow the Herring A Project Evaluation of a Strategic National Tour. Faculty of Arts, Design and Social Sciences.

[B34] Popple K. (2006). Plymouth, paternalism and the astors: the origins of virginia house settlement”, in Drawing on the Past: Studies in the History of Community and Youth Work, eds Gilchrist R. Jeffs T. Spence J. (Leicester: National Youth Agency), 106–122.

[B35] Prentki T. Preston S. (eds.). (2009) Applied Theatre Reader. London; New York, NY: Palgrave.

[B36] Readman G. (2018). [In]visable Identities: an examination of the role of the artistic director in applied theatre making. Drama Res. 9, 1–33.

[B37] Rowbotham S. (1992). Hidden From History: 300 Years of Women's Oppression and the Fight Against it. London: Pluto Classics.

[B38] Spence J. (2019). Twisted seams: a gendered social haunting. J. Work. Class Stud. 4, 5–24.

[B39] Spence J. Stephenson C. (2006). The politics of the doorstep: female survival strategies and the legacy of the miners' strike, 1984-1985. J. Commun. Work Family 10, 309–327. 10.1080/13668800701456252

[B40] Spence J. Stephenson C. (2007). Female involvement in the miners' strike of 1984/5: trajectories of activism. Sociol. Res. 12, 1–11. 10.5153/sro.1461

[B41] Spence J. Stephenson C. (2009). Side by side with our men? Women's activism, community and gender in the 1984-5 british miners' strike'. Int. Labor Work. Class History 75, 68–84. 10.1017/S0147547909000064

[B42] Spence J. Stephenson C. (2012). Pies and essays: women writing through the British 1984–1985 coal miners' strike. Gender Place Cult. 20, 218–235. 10.1080/0966369X.2012.674926

[B43] Stephenson C. Stirling J. Wray D. (2014). Dig where you stand: working life biographies as a challenge to the neo-liberal classroom. Capital Class 38, 399–412. 10.1177/0309816814533173

[B44] Stephenson C. Stirling J. Wray D. (2016). Working lives: the use of auto/biography in the development of a Sociological Imagination. McGill J. Educ. 50, 161–180. 10.7202/1036111ar

[B45] Stephenson C. Wray D. (2005). Emotional regeneration and community action in post industrial mining communities. Miners Strike 87, 175–201. 10.1177/030981680508700111

[B46] Stephenson C. Wray D. (2017). The Gala that would not die: Memory and heritage in the post-industrial mining communities of County Durham” in Justice Denied, Friends, Foes and the Miners' Strike, eds D. Allsop Stephenson C. D. Wray D (London: Merlin Press, 181–194.

[B47] Thomas G. M. (2015). It's not that Bad: Stigma, health and place in post-industrial communities. Health Place 38, 1–7. 10.1016/j.healthplace.2015.12.001 26796322

[B48] Thompson J. Jackson A. (2006). Applied theatre/drama: an e-debate in 2004: viewpoints”. Res. Drama Educ. 11, 90–95. 10.1080/13569780500437960

[B49] Tyler I. (2014). Revolting Subjects: Social Abjection and Resistance in Neoliberal Britain. London: Zed Books.

[B50] Vygotsky L. S. (1986). Thought and Language. Cambridge, MA: MIT Press.

[B51] Waddington D. Critcher C. Dicks B. (2001). Out of the Ashes? The Social Impact of Industrial Contraction and Regeneration on Britain's Mining Communities. London: Routledge.

[B52] Waddington D. Wykes M. Critcher C. (1991). Split at the Seams? Community, Continuity and Change after the 1984-1985 Miners' Strike. Bury St Edmunds: Open University Press.

[B53] Williams R. (2008). “Changing constructions of identity: fisher households and industry restructuring”, Newcastle University (Unpublished Ph.D. thesis). University of Newcastle upon Tyne, Newcastle upon Tyne, United Kingdom.

[B54] Williams Z. (2012, March 9). The Awkward Truth: the class struggle didn't end”. The Guardian.

[B55] Wilson N. (2010). Social creativity: re-qualifying the creative economy. Int. J. Cult. Policy 16, 367–381. 10.1080/10286630903111621

[B56] Wray D. Stephenson C. (2012). Standing the gaff: Immiseration and its consequences in the de-industrialized mining communities of Cape Breton Island”. Capital Class 36, 323–339. 10.1177/0309816812437925

[B57] Wright Mills C. (1959). The Sociological Imagination. Oxford: Oxford University Press.

